# Identification of Circulating Genomic and Metabolic Biomarkers in Intrahepatic Cholangiocarcinoma

**DOI:** 10.3390/cancers11121895

**Published:** 2019-11-28

**Authors:** Helen Winter, Pamela J. Kaisaki, Joe Harvey, Edoardo Giacopuzzi, Matteo P. Ferla, Melissa M. Pentony, Samantha J.L. Knight, Ricky A. Sharma, Jenny C. Taylor, James S.O. McCullagh

**Affiliations:** 1 National Institute for Health Research (NIHR) Oxford Biomedical Research Centre, Wellcome Centre for Human Genetics, University of Oxford, Oxford OX3 7BN, UK; Helen.Winter@UHBristol.nhs.uk (H.W.); pkaisaki@well.ox.ac.uk (P.J.K.); edg1983@well.ox.ac.uk (E.G.); matteo@well.ox.ac.uk (M.P.F.); nicantaine@gmail.com (M.M.P.); jenny@well.ox.ac.uk (J.C.T.); 2 NIHR Oxford Biomedical Research Centre, Department of Oncology, University of Oxford, Oxford OX3 7DQ, UK; ricky.sharma@ucl.ac.uk; 3 Bristol Cancer Institute, Horfield Rd, Bristol BS2 8ED, UK; 4 Chemistry Research Laboratory, Department of Chemistry, University of Oxford, Oxford OX1 3TA, UK; joe@joeharvey.co.uk; 5 NIHR University College London Hospitals Biomedical Research Centre, UCL Cancer Institute, University College London, London WC1E 6DD, UK

**Keywords:** Intrahepatic cholangiocarcinoma, circulating DNA, metabolomics, 2-hydroxyglutarate, IDH1, orotic acid, CAD, DHODH, UMPS, TYMS

## Abstract

Intrahepatic cholangiocarcinoma (ICC) is an aggressive cancer arising from the bile ducts with a need for earlier diagnosis and a greater range of treatment options. *KRAS/NRAS* mutations are common in ICC tumours and 6–32% of patients also have isocitrate dehydrogenase 1 and 2 (*IDH1* and *IDH2*) gene mutations associated with metabolic changes. This feasibility study investigated sequencing circulating tumour DNA (ctDNA) combined with metabolite profiling of plasma as a method for biomarker discovery in ICC patients. Plasma was collected from four ICC patients receiving radio-embolisation and healthy controls at multiple time points. ctDNA was sequenced using Ampliseq cancer hotspot panel-v2 on Ion Torrent PGM for single nucleotide variants (SNV) detection and with Illumina whole genome sequencing for copy number variants (CNV) and further targeted examination for SNVs. Untargeted analysis of metabolites from patient and control plasma was performed using liquid chromatography coupled with high-resolution tandem mass spectrometry (LC-MS/MS). Metabolite identification was performed using multi-parameter comparisons with analysis of authentic standards, and univariate statistical analysis was performed to identify differences in metabolite abundance between patient and control samples. Recurrent somatic SNVs and CNVs were identified in ctDNA from three out of four patients that included both *NRAS* and *IDH1* mutations linked to ICC. Plasma metabolite analysis revealed biomarker metabolites associated with ICC and in particular 2-hydroxyglutarate (2-HG) levels were elevated in both samples from the only patient showing a variant allele in *IDH1*. A reduction in the number of CNVs was observed with treatment. This study demonstrates that ctDNA and metabolite levels can be identified and correlated in ICC patient blood samples and differentiated from healthy controls. We conclude that combining genomic and metabolic analysis of plasma offers an effective approach to biomarker identification with potential for disease stratification and early detection studies.

Intrahepatic cholangiocarcinoma (ICC) is an aggressive cancer arising from the bile ducts with a need for earlier diagnosis and a greater range of treatment options. *KRAS/NRAS* mutations are common in ICC tumours and 6–32% of patients also have isocitrate dehydrogenase 1 and 2 (*IDH1* and *IDH2*) gene mutations associated with metabolic changes. This feasibility study investigated sequencing circulating tumour DNA (ctDNA) combined with metabolite profiling of plasma as a method for biomarker discovery in ICC patients. Plasma was collected from four ICC patients receiving radio-embolisation and healthy controls at multiple time points. ctDNA was sequenced using Ampliseq cancer hotspot panel-v2 on Ion Torrent PGM for single nucleotide variants (SNV) detection and with Illumina whole genome sequencing for copy number variants (CNV) and further targeted examination for SNVs. Untargeted analysis of metabolites from patient and control plasma was performed using liquid chromatography coupled with high-resolution tandem mass spectrometry (LC-MS/MS). Metabolite identification was performed using multi-parameter comparisons with analysis of authentic standards, and univariate statistical analysis was performed to identify differences in metabolite abundance between patient and control samples. Recurrent somatic SNVs and CNVs were identified in ctDNA from three out of four patients that included both *NRAS* and *IDH1* mutations linked to ICC. Plasma metabolite analysis revealed biomarker metabolites associated with ICC and in particular 2-hydroxyglutarate (2-HG) levels were elevated in both samples from the only patient showing a variant allele in *IDH1*. A reduction in the number of CNVs was observed with treatment. This study demonstrates that ctDNA and metabolite levels can be identified and correlated in ICC patient blood samples and differentiated from healthy controls. We conclude that combining genomic and metabolic analysis of plasma offers an effective approach to biomarker identification with potential for disease stratification and early detection studies.

## 1. Introduction

Intrahepatic cholangiocarcinoma (ICC) is a rare cancer, and the second most common primary malignancy of the liver. It arises predominantly as an adenocarcinoma in the bile duct within the liver. Early diagnosis of ICC is currently challenging and only 15% of patients present with resectable tumours with a median survival of up to 3 years [1]. For the majority of patients presenting with advanced disease, the survival remains dismal, with a median survival of less than 12 months [2]. The standard systemic treatments for advanced disease remain platinum-based doublet chemotherapy but many trials have failed to show an increase in overall survival, despite some increased responses [3,4]. Liver-directed therapies, including hepatic arterial infusion (HAI) and selective internal radiation therapy (SIRT), aim at improving disease control. However, little impact on survival has been made and new treatment strategies are urgently needed [5,6]. Biomarkers for earlier diagnosis, early assessment of treatment response and identification of potential novel targets remain important unmet clinical needs. 

Molecular profiling of ICC is a recent area of research focus, with a number of studies attempting to detect therapeutic targets and identify sub-populations which may benefit from a targeted therapeutic approach [7,8,9,10]. For example, in one study, molecular profiling of tissue DNA from 67 ICC patients was performed, with mutations being detected in 25% of patients [11]. Mutations reported included common single nucleotide variants (SNVs) in *KRAS* (G12A, G12D, Q61H); *PIK3CA*; *MET* (T9921, R970C); *BRAF* (V600E); *EGFR* (G719S), and *NRAS* (Q61R), as well as variants in *IDH1, IDH2*, *PTEN*, and *TP53*. Copy number variants (CNVs) have also been reported, including copy number (CN) gains (1p, 1q, 6q, 7p, 11q, 12q) and CN losses (3p, 6q, 8p 9p, 10q, 12p, 12q, 13q, 14q, 16q, 19p, 21q), as have structural variants such as translocations, causing the formation of fusion genes [8,9] involving *FGFR2, ROS1* and *NTRK1* [12,13]. These fusion events may differentiate ICC from other forms of cholangiocarcinoma.

ICC-associated SNVs are linked with a number of signal transduction pathways and oncogenic mechanisms: BRAF, EGFR, KRAS and NRAS proteins are involved in the Ras/Raf/MEK/ERK pathways and common proto-oncogenes [9]. PTEN and PIK3CA are associated with the PI3K/mTOR pathway, which is a target for the mTOR inhibitor, everolimus, now approved in renal clear cell carcinoma [9]. Of particular interest for ICC is the association with isocitrate dehydrogenase (*IDH*) mutations leading to the production of 2-hydroxyglutarate (2-HG).

Mutations in isocitrate dehydrogenase 1 (*IDH1*) were first identified in exome sequencing analysis of patients with colorectal cancer [14]. Hotspot mutations in *IDH1* and *IDH2* have subsequently been shown to occur in at least 13 types of cancer, including 70% of malignant gliomas, 30% of AML and 5–25% of cholangiocarcinomas [5,9,10,11,15,16,17,18,19]. Somatic mutations are frequently found at amino acid position 132 of IDH1, such as R132C and R132H, in the catalytic domain of the protein [18]. These mutations have been shown to reduce the ability of IDH1 to decarboxylate isocitrate and lead to an unusual change in enzyme function. This neomorphic activity leads to production of large quantities of 2-hydroxyglutarate (2-HG), now considered an ‘oncometabolite’ [20] and a potentially important biomarker of *IDH*-mutated tumours. Non-invasive imaging studies, using MRS detection of 2-HG, have been performed on live patients with low–medium grade gliomas [21,22]. 2-HG is known to inhibit histone demethylation, resulting in the accumulation of histone H3K9 marks and increased DNA methylation. Increased 2-HG is also strongly associated with inhibition of at least some 2-oxoglutarate-dependent dioxygenase enzymes [23,24]. IDH is therefore of emerging interest as a drug target, and intensive studies to develop small molecule inhibitors of mutant IDH enzymes have led to a number of anti-cancer therapeutics [25,26,27]. Early clinical data from the application of IDH inhibitor treatment in AML showed a 38% response leading to complete remission of symptoms [28,29]. Application to glioma cells led to mixed results, with some studies achieving a reduction in abundance of 2-HG which was not associated with inhibition of cell growth or tumour progression [30]. Clinical programs are underway to test IDH inhibitors in ICC, for example, AG-221 [26]. *IDH1* mutations producing 2-HG have been found to make ICC cell lines more sensitive to an anti-cancer inhibitor of bromodomain and extraterminal domain (BET) proteins [19]. 2-Hydroxyglutarate has also be shown to create a homologous recombination defect that sensitizes cancer cells to poly(adenosine 5′-diphosphate-ribose) polymerase (PARP) inhibitors, which could serve as another treatment option for these patients [23].

Several studies have looked at ctDNA in patients with ICC [31,32,33] but few have combined this with metabolite analysis. As the ICC tumours are often inaccessible, the ability to measure *IDH* mutations and metabolic markers in plasma has potential to help identify and stratify tumours, as well as determine and monitor therapeutic interventions. As a proof of principle study, we have investigated the feasibility of combining genetic profiling of ctDNA with identification of metabolic biomarkers in blood from ICC patients and healthy controls. We performed analysis of ctDNA, testing for genetic mutations (including single nucleotide and copy number variants), and combined this with untargeted metabolomics.

## 2. Results

### 2.1. Sequencing of Circulating Tumour DNA from ICC Patients

ctDNA and germline DNA (gDNA) were extracted from blood samples taken from each patient at baseline and 4 and 10 weeks after selective internal radiation therapy (SIRT). See Supplementary Table S1 for patient information. Targeted sequencing of ctDNA using a 50 cancer gene hotspot panel (described in Hamblin et al. 2017 [34]) identified two somatic mutations. Three of the four patients were found to have the same mutation in *NRAS* (Q61R) and one patient also had an *IDH1* R132C mutation (Table 1). Total depth of coverage of the *NRAS* and *IDH1* amplicons in the four patients ranged from 4915 to 10,000 reads (the Ion Reporter workflow downsampled to 10,000 reads when depth of coverage exceeded this number).

Whole genome sequencing (WGS) was also performed on the ctDNA and gDNA. Unfortunately, the depth of sequencing coverage was too low to conduct a hypothesis-free, genome-wide analysis of SNVs from ctDNA, although analysis of selected genes was performed (see below). In the ctDNA, coverage ranged from 7–9x, which allowed detection of several copy number variants (CNVs) in three of the four patients, detected by a log-R ratio outside of the normal range of ± 1 (Supplementary Figure S1, Supplementary Table S3) and confirmed by inspection of the sequence in Integrative Genomics Viewer. The depth of WGS sequencing coverage in gDNA ranged from 17.5–18.3x.

At baseline before SIRT, Patient 1 had CN gains on chr. 1, 5, 8 and 12, and losses on chr. 1,3,6,12,13,14,16,17) (Supplementary Figure S1a). The CN loss on chr. 3p, which contains *BAP1* and *PBRM1* genes, is a recurrent CNV and has been observed previously in about 20% of ICC cases [35]. Adjacent to this was another CN loss on 3p containing the tumour suppressor *FHIT*, which has been observed in 60–70% of cholangiocarcinoma patients [36].

CN losses at baseline in chr. 3p containing *BAP1* and *PBRM1* were also observed in Patients 2 and 3, and Patient 3 also had a CN loss involving *FHIT* (Supplementary Figure S1c,f). Patient 2 also had a focal CN loss on chr. 9:21.09–21.98 Mb, which overlaps the last exon of the tumour suppressor gene, *CDKN2A* (Cyclin Dependent Kinase Inhibitor 2A; Supplementary Figure S2a). Patient 3 also had a focal CN loss on chr. 9, from 21.7–22.4 Mb, which includes *CDKN2A*. Loss of *CDKN2A* is associated with progression to cancer [37] (Supplementary Figure S2b). Focal deletions at chromosome 9p21.3 have been observed in 7–18% of ICC patients [8].

Whilst no SNVs were detected in Patient 4 plasma, this patient did, however, have a CN gain of approximately 226kb on chr. 17, a region which included three genes: *USP32* (ubiquitin specific peptidase 32), known to be overexpressed in breast cancer and colorectal metastatic disease [38], *C17orf64* (an open reading frame) and *APPBP2* (amyloid beta precursor protein binding protein 2), also highly expressed in breast cancer [39].

### 2.2. Changes in Variants following SIRT Radiotherapy Treatment

All patients had previously received palliative gemcitabine and cisplatin as standard of care chemotherapy prior to blood sampling. Three (Patients 1,2,3) of the four patients also received SIRT after the baseline blood sample was taken. Patient 1, who at baseline had quite extensive CN variation across the genome (Supplementary Figure S1a), showed a reduction in CNVs following treatment with SIRT (Supplementary Figure S1b). After SIRT, the chr. 3p CN loss, and most other CNVs were no longer detected (Supplementary Figure S1b). The reduction in CN variation was observed to a lesser extent in Patient 2 (Supplementary Figure S1c–e) and Patient 3 (Supplementary Figure S1f,g). It is presumed that less CN variation in the ctDNA is a reflection of a reduced number of tumour cells carrying those mutations following SIRT.

The focal deletions on chromosome 9 of Patients 2 and 3 provided a higher resolution view (Supplementary Figure S2). In both patients, a lower percentage of CN variation in the weeks following SIRT was observed, though not a complete resolution of the mutation.

### 2.3. 2-hydroxyglutarate Abundance Correlates with ctDNA IDH1 Status

**Figure 1:**
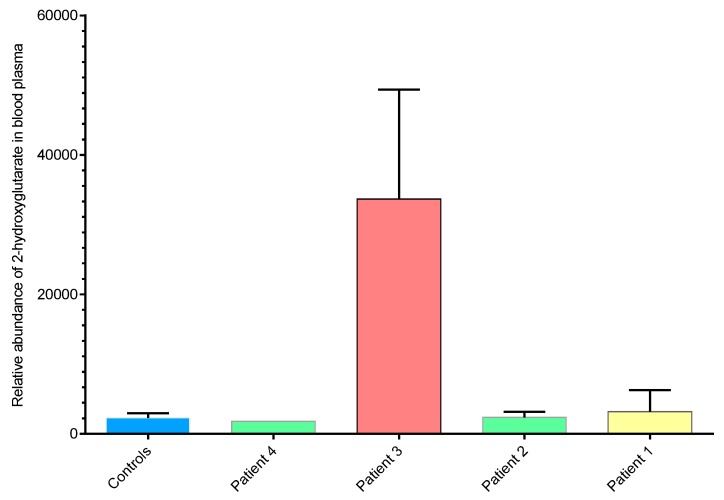
Normalised, relative abundance of 2-hydroxyglutarate (2-HG) levels in blood plasma samples comparing intrahepatic cholangiocarcinoma (ICC) patients and controls. 2-HG was elevated in Patient 3 plasma samples at multiple time points and the same blood samples were positive for circulating isocitrate dehydrogenase 1 (IDH1) mutant DNA. Patients 1, 2, and 4, for whom there were no evidence of IDH mutations, showed similar levels of 2-HG to those of controls (*n* = 8 for controls, *n* = 2 for patient 1, *n* = 2 for patient 2, *n* = 2 for patient 3 (IDH1 mutant), *n* = 1 for patient 4; error bars show standard deviation from the mean).

**Figure 2:**
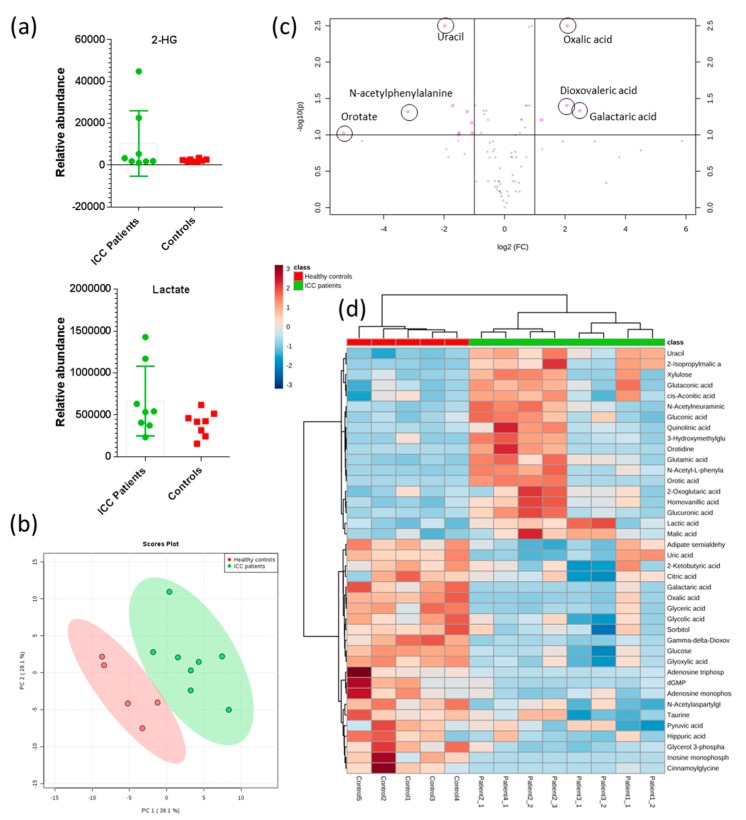
Univariate and multivariate statistical analysis of untargeted metabolomics data comparing ICC vs. control samples identified significant differences in specific metabolite abundances between the two groups: (**a**) Box plots show differences between normalised compound abundances illustrating 2-HG and lactate both correlate directly with *IDH1* mutation status. Both 2-HG and lactate have higher abundance in patient samples with circulating *IDH1* mutation (box plots: *n* = 8 ICC patient samples, *n* = 6 controls, boxes extend from the 25th to the 75th percentile with the median line in the middle. Whiskers are min to max with all data points shown). (**b**) An unsupervised PCA scores plot shows separation of patient samples from controls (95% confidence regions shaded). (**c**) Volcano plot combines fold-change (threshold of 2) and t-tests statistics (FDR adjusted *p*-value cutoff of 0.1 and fold change >2). Data normalised by sum and unequal group variance assumed. (**d**) Hierarchical clustering of the top 35 identified metabolites highlighted a two group trend within the ICC patient cohort. A number of organic acids and N-acetylated amino acids associated with central carbon metabolism were elevated in Patients 1 and 2 compared to Patients 3 and 4.

In light of the *IDH1* mutations identified in Patient 3 samples, we sought to determine the relative levels of 2-hydroxyglutarate (2-HG) and other metabolites in plasma samples. Metabolomics analysis of plasma extracts was performed using anion-exchange chromatography coupled directly to tandem mass spectrometry [40]. Plasma samples from each of the four ICC patients, taken at each time point, were analysed, as well as plasma from six healthy volunteers for comparison. Analysis of authentic metabolite standards was used to identify 79 metabolites, including 2-HG, from the untargeted dataset by matching accurate mass (<5ppm), retention time (<30sec) and isotope abundance patterns (>90%). Figure 1 compares the normalised abundances of 2-HG in the plasma samples extracted from each ICC patient. Plasma samples from Patient 3, (positive for circulating *IDH1* mutation R132C) had elevated levels of 2-HG compared with ICC patients not harboring the mutation and controls. 2-HG levels therefore correlated directly with ctDNA *IDH1* mutation status. No significant differences in 2-HG levels were observed between ICC patients without the *IDH1* R132C mutation and healthy controls. Lactate levels correlated with 2-HG, with elevated levels in Patient 3, for whom a somatic *IDH1* mutation was identified (Figure 2a and Supplementary Figure S3a).

### 2.4. Untargeted Metabolomics Reveals Altered Metabolite Profiles in ICC Patients 

**Figure 3:**
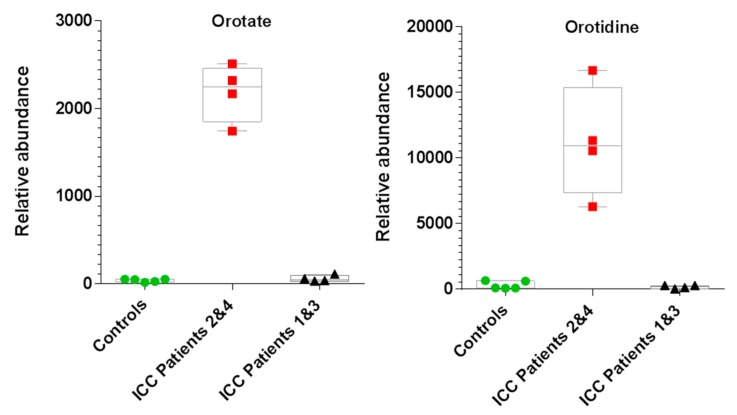
Elevated orotate and orotidine in two ICC patients (Patients 2 and 4) determined by univariate analysis (T-test) comparing liquid chromatography coupled with high-resolution tandem mass spectrometry (LC-MS/MS) extracted ion chromatogram peak areas. Box plots: *n* = 5 controls, *n* = 4 (Patients 2 and 4), *n* = 4 (Patients 1 and 3). Boxes extend from the 25th to the 75th percentile with the median line and whiskers min to max with all data points shown.

Untargeted metabolomics on blood plasma from ICC patients and controls at each time point were performed using the same IC-MS/MS method. Data were processed and analysed using Progenesis QI (Waters LTD, Elstree, UK) for small molecule analysis and MetaboAnalyst [41]. An unsupervised principal component analysis (PCA) scores plot in Figure 2b shows ICC patient and control groups clustered separately. Unsupervised hierarchical clustering (Euclidean distance measure and Ward clustering algorithm), associated with all compound features, grouped samples correctly into their experimental groups (Supplementary Figure S3b), demonstrating both elevated and depleted metabolites in ICC patient samples compared to healthy controls. A volcano plot (log_10_ *p*-value vs. log_2_ fold-change), representing all 82 identified metabolites, revealed that several metabolites were significantly altered in ICC patient plasma samples (Figure 2c and Supplementary Figure S3d). Of these, the greatest fold change between experimental groups was for orotate, which was significantly elevated in ICC patient samples (fold-change 39, *p*-value 0.006). Orotate is an intermediate in the pyrimidine biosynthesis pathway (Supplementary Figure S4). Interestingly, orotate levels could be divided into two groups within the ICC patient cohort: those where orotate levels were significantly elevated (Patients 2 and 4) and those which showed similar levels to healthy controls (Patients 1 and 3). Figure 3 compares orotate and orotidine levels from patient 2 and 4 with those of controls and patients 1 and 3. Seven additional metabolites correlated >90% with the binary orotate profile (Supplementary Figure S3c).

These differences in the metabolic profile suggested the possible presence of causal genetic variants. We therefore re-examined our WGS data for possible SNV and CNV mutations affecting four genes of the pyrimidine biosynthesis pathway (Supplementary Figure S4): the tri-functional enzyme *CAD* (carbamoyl-phosphate synthetase 2, aspartate transcarbamylase, and dihydroorotase), *DHODH* (dihydroorotate dehydrogenase), the bi-functional enzyme uridine 5′-monophosphate synthase (*UMPS*, comprised of orotate phosphoribosyltransferase and OMP decarboxylase), and thymidylate synthetase (*TYMS*). Both CAD and DHODH act upstream of orotate, UMPS uses orotate as a substrate, and, if TYMS is inhibited, it could result in accumulation of orotate through degradation of dUMP (Table 2). Patient 2, who had elevated orotate and orotidine, had several variants that, when combined, may explain the accumulation of these metabolites. These include a trisomy CNV gain of chromosome 2 where *CAD* resides (an increase in CAD protein could cause increased production of orotate precursors), and a monosomy CNV loss of chromosome 3, where *UMPS* is located, which may mean less orotate and orotidine are used, due to reduction in the enzyme, and lead to accumulation of these two metabolites (it was noted, however, that the *UMPS* CNV was also observed in Patient 1, who did not have elevated orotate). Patient 2 also had a germline SNV in *CAD* (V2115L), which is a variant of unknown significance but is located in a functional domain (aspartate/ornithine transcarbamoylase domain). The other patient with elevated orotate and orotidine, Patient 4, had four SNVs in three of the genes we examined: a germline variant in *TYMS*, G5S, and three somatic mutations, *CAD*, C92R; *CAD*, D2047fs; and *DHODH*, A375S. Again, these are variants of unknown significance, but they are rare in the general population (< 1% allele frequency in ExAC and gnomAD databases). 

The presence of two metabolic profiles within the ICC cohort was indicated in the unsupervised hierarchical clustering of identified metabolites (see heatmap in Figure 2d). These data suggested that at least two genetic and/or pathological sub-groups may be present within the ICC cohort. The circulating *NRAS* mutations split the metabolic groups, and the *IDH1* mutation was only found in Patient 3, however, it cannot be ruled out that a more detailed genomic analysis of the tumour itself could have revealed additional mutations which correlated with the metabolic changes observed. Unfortunately, it was not possible to explore this further as there was no access to the tumour tissue itself in this study. The results nevertheless highlight that ctDNA can provide a relevant and straightforward way to identify and stratify ICC patients and that metabolomics analysis can reveal altered metabolic phenotypes in plasma that both correlate with ctDNA mutation status, and may serve to indicate additional genetic and/or clinical differences of potential relevance in early detection studies.

## 3. Discussion

We measured circulating tumour DNA and demonstrated the presence of SNVs and CNVs in samples taken from ICC patients and linked this to plasma metabolite analysis. The 50 cancer gene panel detected an *IDH1* mutation in one of the four patients (Patient 3). This is in good accordance with the reported range of incidence of *IDH1* mutations in ICC (5–25%) [5,9,10,11,19]. Metabolomics analysis revealed that this patient had significantly elevated plasma 2-HG (measured at multiple time points) compared with other ICC patients without *IDH1* mutations and healthy controls. This corresponded with the results of other research where circulating 2-HG levels were predictive of *IDH1* mutations in tissue [42,43]. Circulating 2-HG has been proposed as a potential biomarker in patients with *IDH-*mutant ICC and our results here are commensurate [42]. A phase I clinical trial of the IDH1 inhibitor AG-220 (NCT02073994 Agios Pharmaceuticals) is currently underway for a range of solid tumours, including ICC, to investigate the safety, pharmacodynamics and pharmacokinetics of this inhibitor. Such clinical trials are important to assess whether the inhibitor is effective in this cancer type, as results have been variable in other cancer types. IDH inhibitors have been shown to be effective in AML in depleting 2-HG, resulting in partial or substantial remission for a significant proportion of patients. However, although in vitro studies of the IDH1 inhibitors in gliomas have demonstrated a reduction in 2-HG, this has not been linked to response. 

Additional metabolites associated with central carbon metabolism were also elevated in plasma samples from our ICC patients and hierarchical clustering showed that ICC patients could be split into two groups based on their metabolite profile. Orotate (pyrimidine pathway) was significantly elevated at each time point in two out of four ICC patients (Patients 2 and 4, Figure 3 and Supplementary Figure S3) and a similar profile was also observed for other metabolites (e.g., N-acetylphenylalanine; glucoronate; 2-hydroxymethylglutarate; N-acetylnuraminate and quinolinate), but not for the majority of identified metabolites. Genes associated with orotate biosynthesis are not recognised as clinically actionable cancer genes and were therefore not included in the 50 cancer gene panel. However, we had obtained WGS data from gDNA and ctDNA samples, and, although coverage was too low to confidently designate SNVs on a genome wide basis, we were able to inspect the WGS data for SNVs and CNVS in the corresponding genes/regions involved in pyrimidine biosynthesis for our ICC cohort. 

We identified a germline SNV in the *CAD* gene (V2115L) in Patient 2 (Supplementary Table S3). CAD is a hexameric protein and each polypeptide chain is composed of three domains, each encoding a different enzymatic step in the pyrimidine biosynthesis pathway (CPS2, DHOase, ATCase). The SNV V2115L, whilst a variant of uncertain significance, is in the ATCase domain of the protein, and therefore might have a functional effect. Based on protein structural predictions (https://michelanglo.sgc.ox.ac.uk/r/cad), the variant may affect the co-operative binding behavior and could possibly interfere with the transition from active to inactive state [44]. In addition, this patient also had a trisomy of chromosome 2, where CAD resides, which could predict increased levels of CAD activity that could account for the increased levels of orotate in plasma observed in Patient 2. 

Similarly, Patient 4, who also had elevated orotate levels, had four SNVs in three genes of the pyrimidine biosynthesis pathway that we examined, including two somatic heterozygous mutations in *CAD* (C92R and D2047fs) and a somatic variant in *DHODH* (A375S) as well as a mild germline variant in TYMS (G5S). The *CAD* C92R variant may interfere with allosteric regulation and possibly result in a gain of function phenotype (Supplementary Table S3). The *CAD* D2047 frameshift variant lies within the active site of the enzyme (residues R2024-Q2148) [44]. With a lack of nonsense-mediated decay, truncation may be expected to lead to reduced substrate binding in the mutant chain and loss of function, i.e., reduced, not increased levels of orotate. However, the D2047fs is in the latter half of the ATCase domain of *CAD*, which is activated by co-operative binding [44]. It is feasible that the truncated chain may still participate in oligomerisation and have a positive influence on the activation state of the wild type enzyme (https://michelanglo.sgc.ox.ac.uk/r/cad). In this scenario, overall CAD activity could be increased by the frameshift mutation, resulting in higher orotate. Our frameshift mutation is a somatic mutation present at very low frequency and functional studies would be required to assess its actual effect on orotate levels, whether it acts in combination with the C92R variant, and whether the wild type allele alone is sufficient for activity. 

This patient also had a mutation in *DHODH.* Notably, compound heterozygous mutations in this gene were identified as the cause of Miller syndrome in the first application of exome sequencing for diagnosis of a rare Mendelian disorder [45]. Analysis of further patients with *DHODH* mutations showed a loss of DHOdhase activity but paradoxically did not show an increase in the enzyme substrate, DHO, in the urine as expected, but instead elevated levels of orotic acid [46]. The somatic surface mutation A375S in Patient 4 lies between some of the previously reported variants [46]; therefore, whether this variant per se results in raised orotate merits further investigation. 

The raised orotate levels observed in Patients 2 and 4 may therefore be explained by the germline or somatic variants in enzymes of the pyrimidine biosynthesis pathway. We cannot differentiate from our data whether these are involved in carcinogenesis, or are secondary effects of this, or, indeed, are bystander effects. Experimental studies in rats indicate that orotic acid promotes liver carcinogenesis, when tumours are initiated by other agents such as 1,2 dimethylhydrazine [47,48,49]. However, protective effects of orotic acid were seen in rats treated with ethionine [50], and in mice treated with methylcholanthrene [51]. Orotic acid has also been shown to promote lung metastases in mice with mammary tumours [52]. Based on these findings, a larger study in ICC patients is indicated to consider whether genetic and metabolomics changes alter the purine/pyrimidine balance and cellular proliferation in the liver, thereby contributing to the pathogenesis of ICC. 

The alterations in orotate are therefore intriguing; elevated orotate levels have been reported in selected cell lines after treatment with the anti-cancer drug 5-fluorouracil (5-FU) [53]. This drug is metabolized by orotate phosphoribosyltransferase (OPRT), the enzyme which also converts orotate to orotidine monophosphate in vivo [54,55]. OPRT converts 5-FU to its active metabolite fluorouridine monophosphate. Because both orotate and 5-FU are substrates for OPRT, it is possible that pharmacological doses of 5-FU could out-compete orotate as a substrate, and lead to accumulation of orotate. However, all patients in this study had received chemotherapy with cisplatin and gemcitabine with no 5-FU treatment, hence 5-FU cannot be an explanation for the increase in orotate observed. It is possible that the increase in orotate may be explained by the variants we identified in pyrimidine biosynthesis genes, although further functional investigation would be required to confirm this conjecture. 

Recently interest has returned to using nucleotide synthesis as a metabolic vulnerability in cancer cells [56]. Inhibitors of pyrimidine synthesis may target tumour progression, and can sensitise cancer cells to other forms of chemotherapy [56]. 5-Fluorouracil (5-FU) is a well-known example of an anti-cancer drug targeting this pathway [53]. The anti-tumour drug N-phosphonacetyl-L-aspartate (PALA) targets a domain of CAD in pyrimidine biosynthesis [44]. Inhibition of pyrimidine biosynthesis with 5-aminoimidazole-4-carboxamide-1-β-riboside (AICAr) induces apoptosis in multiple myeloma cells, with inhibition of UMPS and increases in orotate, further supporting pyrimidine biosynthesis as a potential molecular target in multiple myeloma cells [57]. It has recently been observed that cholangiocarcinoma patients with high expression of OPRT benefited from postoperative adjuvant chemotherapy with S-1 (an oral fluoropyrimidine, which is an OPRT inhibitor) [58]. It is possible that different genetic variants of the enzymes in pyrimidine/orotate synthesis, such as the variants we have observed, will be more or less sensitive to these treatments, and, therefore, ctDNA sequencing of these genes and orotate detection by metabolomics could help in selecting better personalised treatment for these patients. 

A limitation of this study was the small number of ICC patients and controls that were available for analysis. A further limitation was the 50 cancer gene panel used for targeted sequencing, as it only covered hotspot mutations in the 50 genes most commonly mutated in a broad range of cancers (listed in Supplementary Table S5), whereas the use of a comprehensive and specific upper GI cancer panel, including genes associated with recurrent mutations in ICC such as *ARID1A* and *BAP1* [9], may provide genetic profiling more specific to ICCs. Furthermore, copy number changes remain undetected when using this targeted cancer gene panel, which led us to perform WGS on the ctDNA. Whilst the low levels of ctDNA compromised our ability to accurately call SNVs, we did observe recurrent CNVs on chromosomes 3 and 9. Previous CNV studies have used genomic DNA extracted from FFPE tumour tissue; this is the first time to our knowledge, that these CNVs have been detected in ctDNA from patients with ICC, providing further support for use of ctDNA as a biomarker in ICC. The reduction in the number of CNVs observed after SIRT treatment indicates that that analysis of both CNVs and SNVs in ctDNA pre and post treatment may be a useful clinical marker of response to therapy or progression. Detection of gene fusion events such as those involving *FGFR2* are relatively common in ICC, and the ability to detect these may be important for therapeutic targeting [8]. We examined our WGS data for fusions, but did not find any evidence for them in these patients. It should be noted that this was primarily a feasibility study and, while genetic and metabolic markers associated with the ICC patients are intriguing, further validation of these results in a larger cohort study is indicated.

In summary, we have analysed genetic profiles from ctDNA and plasma metabolomics data from the same ICC patients and found correlations between specific genes and altered metabolite levels, and, for selected gene mutations, direct links to altered metabolites. For example, an increase in 2-HG further to somatic mutation in *IDH1* was found in one of our patients. Results of ongoing clinical trials will be required to assess the efficacy of IDH1 inhibitors in reducing 2-HG levels and improving clinical outcomes for ICC patients with such *IDH1* mutations. We also identified increases in the metabolite orotic acid in two of our patients. Somatic mutations found in the ctDNA may account for this increase. To our knowledge, this is the first time this metabolite has been shown to be elevated in ICC, or, indeed, any cancer type. Further large-scale studies are required to demonstrate the significance of the increase in orotate that we have observed here in a small number of ICC patients and, if replicated, to assess whether orotate contributes to the promotion or induction of carcinogenesis, or whether it is a secondary effect, before this metabolite could be considered as a candidate for targeted therapy. In conclusion, we consider that the combination of ctDNA and metabolomics offers potential for identifying novel biomarkers and to indicate treatments for ICC patients based on their mutation profile, as well as supporting the monitoring of response to therapies or disease progression. 

## 4. Materials and Methods 

### 4.1. Methods and Patients

Four patients provided written informed consent to participate in this clinical trial in patients with liver predominant malignancy. Berkshire Research Ethics Committee trial number: 10 H0505 95. Patient details are provided in Supplementary Information.

### 4.2. Sample Processing and Extraction of ctDNA and gDNA

To minimise lymphocyte lysis, blood samples were centrifuged at 2060× *g* (3000 rpm in Beckman GS-6R centrifuge) for 10 minutes at room temperature without brake within 6 hours of collection [59]. Plasma was transferred to a new tube, mixed, and aliquots were pipetted into microfuge tubes. After 10 min in the centrifuge at 7000 rpm, the supernatants were transferred to new microfuge tubes and stored at −80 °C until DNA extraction. ctDNA was isolated using Qiagen QIAamp Circulating Nucleic Acid kit according to the manufacturer’s protocol (QIAGEN Ltd., Manchester, UK). Matching gDNA was extracted from whole blood using Qiagen QIAamp DSP DNA Blood mini kit. DNA quantity was determined using Qubit dsDNA High Sensitivity assay kit on a Qubit 2.0 Fluorometer (ThermoFisher, Paisley, UK).

### 4.3. Targeted Sequencing Using 50 Cancer Gene Panel

Ion AmpliSeq™ Cancer Hotspot Panel v2 (50 cancer gene panel) was used to detect potentially actionable somatic mutations, using a collection of primers designed to interrogate hotspot regions in 50 genes (207 amplicons) commonly mutated in cancer (ThermoFisher Scientific, Waltham, MA USA). Gene information is listed in Supplementary Table S5. Libraries were generated according to the manufacturer’s protocol. Briefly, multiplex PCR was performed with the Ion Ampliseq library kit 2.0 using approximately 10 ng DNA and the panel primer pool. Following PCR, the amplicons were partially digested and IonXpress-barcoded adapters were attached by ligation. The libraries were purified using Agencourt AMPure XP magnetic beads (Beckman Coulter Ltd., High Wycombe, UK) and amplified using adapter primers. The amplified libraries were purified again, and quantified on an Agilent 2100 Bioanalyzer using the High Sensitivity DNA kit (Agilent Technologies, Stockport, UK). Library templates for sequencing were prepared by emulsion PCR on the One Touch 2 instrument and loaded onto 318 semiconductor chips and the Ion Torrent PGM™ sequencer [60]. Sequence analysis was performed using Torrent Variant Caller (TVC) v4.4.5. Somatic variants were detected using AmpliSeq CHPv2 peripheral/CTC/CF DNA analysis workflow in the Ion Reporter v.5.12.1.0 with standard parameters. The software is optimised for Ion Torrent flow-space data and applies the following main filters during variant identification: minimum genotype quality of 10, minimum coverage of 600, minimum coverage of 10 on each strand, minimum two observations of the alternate allele, minimum allele frequency of 0.005 and 0.007 for SNPs and INDELs, respectively. When depth of coverage exceeded 10,000, the workflow downsampled to 10,000 reads. Only variants passing all filters were considered in downstream analyses. Matching gDNA sequence was inspected for variants detected in a patient’s ctDNA, in order to confirm whether ctDNA variants were somatic, i.e., absent from the germline sequence. 

### 4.4. Library Preparation for whole Genome Sequencing of Plasma DNA Samples

Libraries were prepared from plasma DNA as described previously [61]. Briefly, samples were quantified using the Quant-iT™ PicoGreen ^®^ dsDNA Kits (Invitrogen) and sample integrity was assessed using Agilent’s Tapestation system both in accordance with the manufacturer’s specifications. Libraries were constructed using Wafergen’s PrepX Complete ILMN 32i DNA Library Kit (cat number 400076) with an input of 5 ng where available using the Apollo 324. Ligation of adapters was performed using Illumina Adapters within the Multiplexing Sample Preparation Oligonucleotide Kit. Each library was PCR enriched with 25 µM each of the following custom primers: 

Multiplex PCR primer 1.0: AATGATACGGCGACCACCGAGATCTACACTCTTTCCCTACACGACGCTCTTCCGATCT

Index primer: CAAGCAGAAGACGGCATACGAGAT[INDEX]CAGTGACTGGAGTTCAGACGTGTGCTCTTCCGATCT

Enrichment and adapter extension of each preparation was obtained using 15 µL of size-selected library in a 50 µL PCR reaction using NEBNext High-Fidelity 2X PCR Master Mix. Reactions were purified with Ampure beads (Agencourt/Beckman) on a Biomek NXp. The concentrations used to generate the multiplex pool were determined by Picogreen and pooling was performed using a Biomek 3000. The final size distribution of the pool was determined using a Tapestation 1DK system (Agilent), quantification for sequencing was determined using Qubit. The plasma DNA sample from one ICC patient (Patient 2) and a normal control were also subjected to library preparation using the ThruPLEX^®^ Plasma-seq Kit, according to manufacturer instructions (Rubicon Genomics, U.S.A.).

Plasma DNA libraries were sequenced on an Illumina HiSeq4000 with 75bp, paired-end sequencing.

### 4.5. Library Preparation for whole Genome Sequencing of Genomic DNA Samples

Samples were quantified using the Quant-iT™ PicoGreen ^®^ dsDNA Kits (Invitrogen) and sample integrity was assessed using Agilent’s Tapestation system both in accordance to manufacturer specifications. Samples were normalised to 1 ug before fragmentation using the Covaris platform. Libraries were subsequently prepared using the NEBNext Ultra Library Prep Kit for Illumina (NEB) as per manufacturer’s recommendations, but with the following minor changes: size selection performed on 2% E-Gel; sample was split and four PCRs performed; and custom indexes, as for plasma DNA. The final size distribution of the pool was determined using a Tapestation 1DK system (Agilent), quantification for sequencing was determined using Qubit.

Genomic DNA libraries were sequenced on a HiSeq4000 with 150 bp, paired-end sequencing.

### 4.6. Mapping and Variant Calling in whole Genome Sequence (WGS) Data

Illumina WGS data was base-called with bcl2fastq version 2.17.1.14 (Illumina), pre-mapped using bwa version (v0.7.0 and v0.7.15) [62] against hs37d5, then re-aligned using stampy (v1.0.23 and v1.0.31) [63], and sorted using Picard v1.111 (http://broadinstitute.github.io/picard/). Duplicates were marked and removed (also with Picard v1.111). Copy number variation detection was performed on WGS data using Nexus software (BioDiscovery, Inc.). Sequence data from each ICC patients’ ctDNA were compared to their own gDNA sequence, for identification of copy number loss or gain. ICC CNVs were also compared to those found in WGS of plasma DNA and gDNA from a healthy control individual, prepared in the same time period using the same protocols and reagents, to provide a control for possible artefacts due to the extraction or sequencing methods.

We investigated the presence of germline and somatic variants in four genes involved in pyrimidine metabolism: CAD, DHODH, UMPS, TYMS. Germline variants in the region corresponding to these four genes were jointly called on all samples using Platypus variant caller v.0.8.1 [64] with default parameters, except for minFlank set to zero value. Given the relatively low coverage of the WGS data, we combined three different algorithms to improve the identification of somatic variants. First, potential somatic variants limited to our target region were identified in each tumor sample comparing tumor and normal BAM files using each one of Strelka2 v.2.9.10 [65], MuTect2 (as implemented in GATK v.3.7) [66] and VarScan2 v.2.3.9 [67]. The somatic variants identified by each algorithm were then merged in a single VCF file for each tumor sample. Variants were manually verified to have supporting sequence reads using the Integrative Genomics Viewer (IGV v.2.3.32, [68]. For both germline and somatic analyses, variants were annotated using Annovar [69] to include gene level annotations, variant frequency from ExAC v.0.3.1 and gnomAD v.2.11 populations, mutation impact prediction scores and COSMIC v89 annotations. Best candidate variants were selected as non-synonymous exonic variants with MAF < 1% in all human populations. Interactive visualisations were created using Michelaɴɢʟo (manuscript in preparation). Conclusions were drawn by observations of several structures, namely the Swiss-Model for CAD 1-1442, based upon PDB 5DOU [70], and the PDB structures 2PRL, 5DOU, 4C6E, 5G1N and 5G1O [44,71,72,73].

### 4.7. Metabolomics by IC-MS/MS 

#### 4.7.1. Metabolite Extraction

All plasma samples were prepared for liquid chromatography mass spectrometry (LC-MS) analysis using a single-phase extraction method. Firstly, plasma samples were thawed and then centrifuged (13,000 rpm) at 4 °C for 30 mins. The resulting supernatant was removed and added to a 1.5 mL Eppendorf tube; 300 µL of this supernatant was added to 700 µL methanol and the mixture was centrifuged again (13,000 rpm) at 4 °C for 30 mins. The resulting supernatant was then divided into two parts; 400 µL was added to a total recovery vial (pre-slit PTFE capped total recovery vial (Waters, Corporation, Elstree, UK) for analysis by LC/MS/MS using a reverse phase (C18) chromatography method. A second 400 µL aliquot of supernatant was filtered using a pre-washed (milli-Q water) 10 kd molecular weight cut-off filter (Amicon ultra 0.5 mL centrifugal filters, Sigma-Aldrich, Gillingham, UK) for analysis by ion chromatography coupled directly to tandem mass spectrometry (IC-MS/MS). The filtered supernatant was transferred to a separate total recovery vial. Both vials, containing filtered and unfiltered samples, were stored at -80 °C until the day of analysis.

#### 4.7.2. IC-MS Analysis

A Thermo Scientific Ultimate ICS-5000 was coupled directly to a Q-Exactive hybrid quadrupole-Orbitrap mass spectrometer with a HESI II probe (QE-MS, Thermo Scientific, San Jose, CA). A 5 μL partial loop injection was used for all analyses and chromatographic separation was performed using a Thermo Scientific Dionex IonPac AS11-HC 2 × 250 mm, 4 μm particle size column with a Dionex Ionpac AG11-HC 4 μm 2 × 50 guard column inline. A hydroxide ion gradient was used as follows: 0 mins, 0 mM; 1 min, 0 mM; 15 mins, 60 mM; 25 mins, 100 mM; 30 mins, 100 mM; 30.1 mins, 0 mM; 37 mins, 0 mM. The flow rate was 0.250 mL/min and the total run time was 37 mins. All analysis was performed in negative ion mode and the relevant source parameters were set as follows for IC: sheath gas flow rate, 60; auxiliary gas flow rate, 20; sweep gas flow rate, 0; spray voltage, 3.6 kV; Capillary temperature, 320 °C; S-lens RF level, 70 and heater temperature 450 °C. Other parameters included: Scan range, 60 to 900 m/z; Resolution, 70,000; maximum injection time (Max IT) 250 ms and automated gain control (AGC) target, 1 × 106 ions.

#### 4.7.3. Data Processing and Analysis

Data Processing: Raw data was processed using Progenesis QI for small molecules (Waters, Elstree, UK). Briefly, this encompassed chromatographic peak alignment, isotope cluster recognition (peak picking) and compound identification. Identification of compounds in experimental samples was based on matching to an in-house library of authentic standards, using four measured parameters for each compound from the database. These were: accurate mass measurement (<5 ppm) based on theoretical mass derived from the chemical formula, experimental retention time window of 0.5 mins, isotope pattern recognition (calculated from chemical formula) and matching with fragmentation patterns from an authentic standard where these were available from survey scans. All measured values in the database were obtained from the analysis of authentic standard compounds. A full list of identified metabolites and their raw extracted ion chromatogram (EIC) peak areas from each sample can be found in Supplementary Table S4.

#### 4.7.4. Statistical Analysis

Statistical analysis was performed using Progenesis QI and the EZ info plugin for Progenesis QI developed by Umetrics. *p*-values, %CV and fold-changes associated with the statistical comparison of experimental groups were calculated for each metabolite using progenesis QI. These data were used to identify and evaluate potential biomarkers. The identified metabolites were sorted, by three different methods, according to the maximum fold-change between: i) healthy volunteers and IDH1 positive ICC patients, ii) healthy volunteers and iii) IDH1 negative ICC patients and IDH1 negative and IDH1 positive ICC patients. Difference in metabolite abundance, which was statistically significant (*p* < 0.05), were mapped onto metabolic pathways in a heat-map format for visualisation.

## 5. Conclusions

In conclusion, the role of liquid biopsies including ctDNA and plasma metabolites may be particularly useful in hard-to-reach, hard-to-treat cancers such as ICC. By combining circulating genomic and metabolite analysis we have been able to provide preliminary data using this approach. Our results suggest this has potential to identify novel genetic markers and inform diagnosis and/or prognosis in ICC. We see scope for a larger cohort study to validate the correlation between circulating metabolites and ICC mutational status, particularly our observation that orotate levels are increased in some patients, potentially as a consequence of genetic variants. This minimally invasive approach for the detection of tumour mutations, with complementary metabolite profiling, may also be useful for early detection and disease monitoring in response to therapy.

## Figures and Tables

**Table 1:** Hotspot mutations detected in circulating tumour DNA of four patients with ICC.

Patient	Baseline Quantity ctDNA (ng/µL)	Mutation (Single Nucleotide Variants (SNV))	VAF (%) Baseline	VAF (%) 4 Weeks Post-SIRT	VAF (%) 10 Weeks Post-SIRT
1	1.71	*NRAS*, NM_002524.5: c.182A > G; p.Q61R	0.6 (T = 5168, C = 31)	na	nd
2	18	*NRAS*, NM_002524.5: c.182A > G; p.Q61R	46 (T = 4408, C = 3773)	28 (T = 6375, C = 2518)	32 (T = 4254, C = 2000)
3	2.98	*NRAS,* NM_002524.5: c.182A > G; p.Q61R	21 (T = 7509, C = 2014)	8 (T = 7819, C = 680)	na
		*IDH1*, NM_005896.3: c.394C > T; p.R132C	12 (G = 8490, A = 1109)	5 (G = 9369, A = 480)	na
4	1.19	None	nd	na	na

VAF—% variant allele fraction with allele counts in brackets. A detection threshold of 0.5% variant allele fraction was applied. na—not available, as Patient 3 deceased and Patient 4 was lost to follow up (Patient 4 did not receive SIRT); nd—variant not detected according to filtering criteria.

**Table 2:** Copy number and single nucleotide variants in pyrimidine metabolism pathway genes.

Gene Symbol	Genomic Location (GRCh37/hg19)	Patient 1		Patient 2			Patient 3		Patient 4		
		CNVs in Region of Gene	SNVs	CNVs in Region of Gene	SNVs	Supporting SNV Reads at Baseline (Mutant/Total Reads)	CNVs in Region of Gene	SNVs	CNVs in Region of Gene	SNVs	Supporting SNV Reads at Baseline (Mutant/Total Reads)
*CAD*	chr2: 27,440,258-27,466,811	none	none	chr2 CN gain (incl *CAD*)	*CAD*, NM_004341: c.G6343C; p.V2115L (germline)	14/27	none	none	none	*CAD*, NM_004341: c.T274C; p.C92R	3/24
										*CAD*, NM_004341: c.6139_6140del; p.D2047fs	4/36
*DHODH*	chr16: 72,042,487-72,058,955	chr16 CN loss (incl *DHODH*)	none	none	none	na	none	none	none	*DHODH*, NM_001361: c.G1123T; p.A375S	2/26
*UMPS*	chr3: 124,449,213-124,468,120	chr3 CN loss (incl *UMPS*)	none	chr3 CN loss (incl *UMPS*)	none	na	none	none	none	none	na
*TYMS*	chr18: 657,604-673,578	none	none	none	none	na	chr18 LOH (incl *TYMS*)	none	none	*TYMS*, NM_001071:c.G13A; p.G5S (germline)	9/24

Whole genome sequence data were examined for SNVs and copy number variants (CNVs) relating to four genes of the pyrimidine metabolism pathway: *CAD* (tri-functional enzyme, carbamoyl-phosphate synthetase 2 (CPS2), aspartate transcarbamylase (ATCase), and dihydroorotase (DHOase)); *DHODH* (dihydroorotate dehydrogenase); *UMPS* (bi-functional enzyme, orotate phosphoribosyltransferase and OMP decarboxylase); and *TYMS* (thymidylate synthase). LOH, loss of heterozygosity; na, not applicable.

## Supplementary Materials

The following are available online at https://www.mdpi.com/2072-6694/11/12/1895/s1: Supplementary Figure S1: Copy number analysis of whole genome sequencing data before and after SIRT; Supplementary Figure S2: Focal chromosome 9 copy number loss in Patients 2 and 3; Supplementary Figure S3: Metabolomics data analysis; Supplementary Figure S4. Pyrimidine synthesis pathway; Supplementary Table 1: Patient information; Supplementary Table S2: Cancer gene abbreviations list; Supplementary Table S3: Sequencing results from circulating DNA of ICC patients. Supplementary Table S4. Full list of identified metabolites and their raw extracted ion chromatogram (EIC) peak areas from all samples. Supplementary Table S5. 50 cancer gene panel information.
