# Supplementary material for: Identification of Circulating Genomic and Metabolic Biomarkers in Intrahepatic Cholangiocarcinoma
Source: Cancers (Basel). 2019 Nov 28;11(12):1895. doi: 10.3390/cancers11121895 (PMC6966597; doi:10.3390/cancers11121895)
Supplement: Supplementary file 1 [file cancers-11-01895-s001.zip › ICC_ctDNA_metabolomics-supplementary figures and tables.docx]

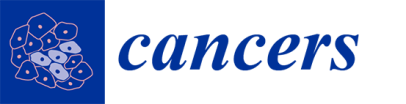


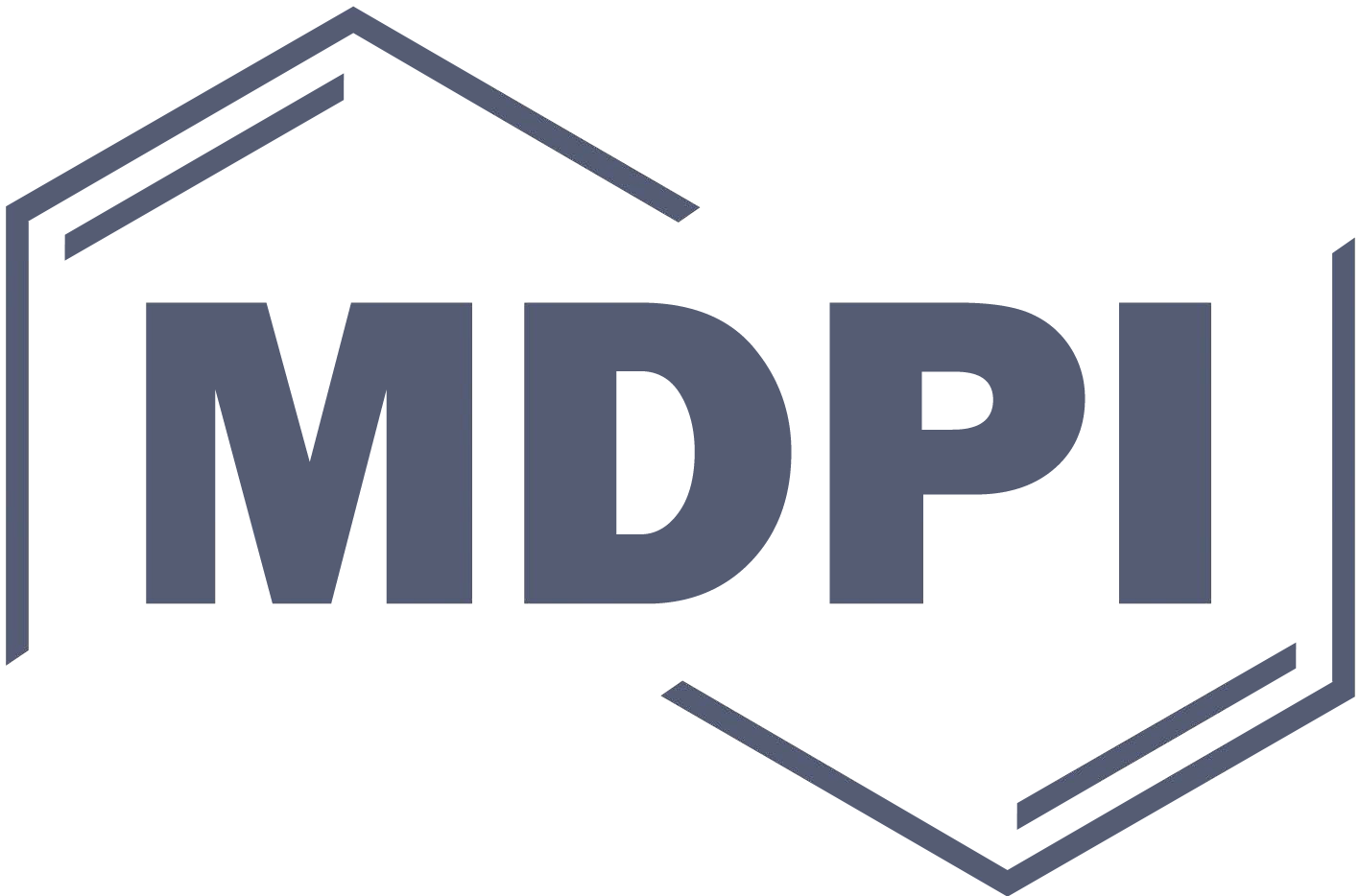


**Identification of circulating genomic and metabolic biomarkers in intrahepatic cholangiocarcinoma**

**Supplementary Materials**

**Supplementary patient information**

Patient 1: Adult female diagnosed through liver biopsy. There was no significant past medical history (PMH) and she remained of an excellent performance status throughout her treatment. She was a lifelong non-smoker, and had hepatitis A as a child. At diagnosis, a liver biopsy confirmed a well to moderately differentiated CK7 positive tumour. Staining for CK 20, CDX2, TTF-1, Napsin-A, GCDFP, ER and PR were all negative. Neither the morphology nor immunoprofile was specific, however the diagnosis was made on findings compatible with cholangiocarcinoma primary. Subsequent molecular profiling of the primary tumour did not identify any hotspot mutations.

She received palliative gemcitabine and cisplatin with a partial response (PR) seen after 4 cycles, in the large intrahepatic bilobar disease. Standard imaging with CT and MRI after chemotherapy confirmed disease progression with increase in size and number of liver lesions. Selective internal radiation therapy (SIRT) delivered 760 MBq and 820 MBq into the anterior and posterior divisions of the right hepatic artery, respectively.

Subsequently, the patient was re-challenged with gemcitabine and cisplatin when she had rising CA 19-9 and increased numbers of bilateral lung nodules. Once disease had progressed within the liver the patient was referred for consideration of Phase I trial. Overall survival from diagnosis was 24 months.

Patient 2: This female was diagnosed with metastatic ICC 12 months prior to SIRT. She had completed six cycles of palliative gemcitabine and cisplatin which was well-tolerated, and stopped for five and a half months before receiving 2000 MBq to the common hepatic artery, using Yttrium 90 (Y-90) resin spheres, for asymptomatic disease progression. Past medical history (PMH) included osteoarthritis with knee replacement, deep vein thrombosis and hypertension. Baseline scan revealed an extensive left sided ICC with multiple small metastases on the right. She required steroids following SIRT for abnormal liver function tests (LFTS) with an albumin that had decreased from 31 to 24 and alkaline phosphatase (ALP) that was raised from 364 to 552, compatible with radiation induced liver disease. Ca19-9 was 403 prior to therapy. Unfortunately, at first follow up scan, 10 weeks after SIRT, she had progressive disease in liver, lung and bone. She died 99 days following SIRT and at 15,5 months after diagnosis.

Patient 3: This female presented with right shoulder and upper quadrant pain and CT scan confirmed a large intrahepatic mass. She had an extended left hepatectomy, which was complicated by liver necrosis and a sub-diaphragmatic collection. Histology confirmed a large ICC measuring 95mm pT2B, N0, L1, V1, and R2 multifocal ICC. Positive immunostaining of tumour cells for CK8/18, CK19, CK7, AE1/AE3 and vimentin and negative immunostaining for CK20, HepPar1 and glypican 3 confirmed cholangiocarcinoma. The tumour showed a few satellite nodules, perineural, lymphatic and vascular invasion. She was previously extremely fit and well and had smoked for 20 pack years. She received “adjuvant gemcitabine and cisplatin” for the R2 margin, which was complicated by an episode of neutropenic sepsis. The first follow up CT scan at 12 weeks, demonstrated a 6cm cystic lesion and new 4mm liver lesion. Liver metastases were confirmed on MRI and chemotherapy continued, with palliative intent. Nine months later imaging confirmed progressive disease (PD) in the liver and enlarging upper abdominal lymph nodes. The patient received SIRT, however continued to deteriorate and died after 41 days from initiation of SIRT. Overall survival from diagnosis was 15 months.

Patient 4: This male was diagnosed with inoperable ICC by laparoscopic biopsy. He had completed gemcitabine and cisplatin before entering the study and blood profiling. He survived 28 months from diagnosis and received two courses of palliative capecitabine and oxaliplatin after baseline blood test.

**Supplementary Figure 1.** Copy number analysis of whole genome sequencing data before and after SIRT (a) Patient 1 at baseline and (b) Patient 1, 10 weeks after SIRT; (c) Patient 2 at baseline, (d) Patient 2, 4 weeks after SIRT, (e) Patient 2, 10 weeks after SIRT; (f) Patient 3 at baseline, (g) Patient 3, 4 weeks after SIRT. Bins of read counts are shown across the genome. Chromosome number shown across the x-axis, and Log R ratios shown on the y-axis. The genes in the CNVs are labelled where prior literature shows involvement of gene or region with ICC [8, 9] . The genes in gains are above in blue, and the losses are in red, below.


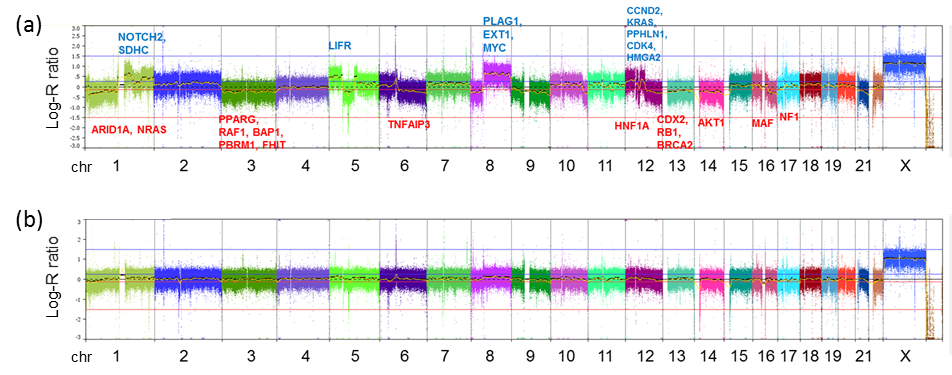

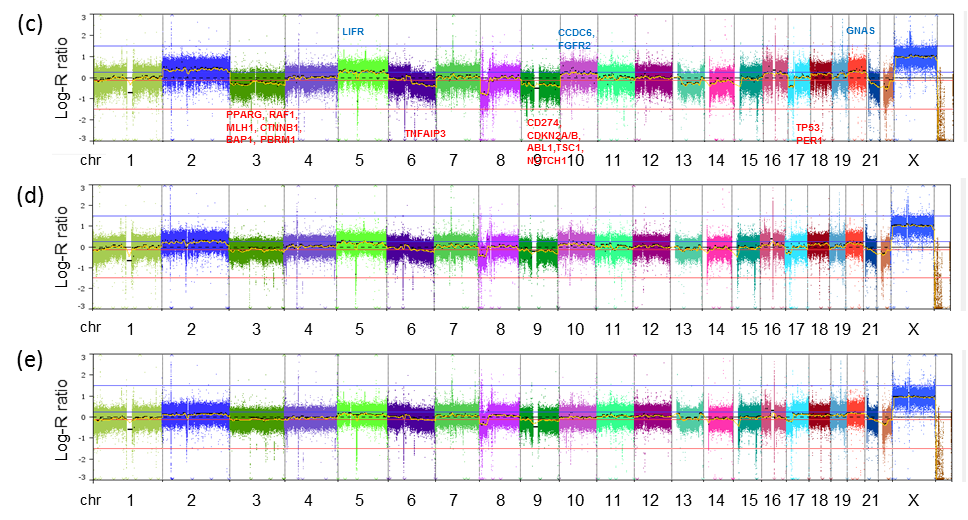

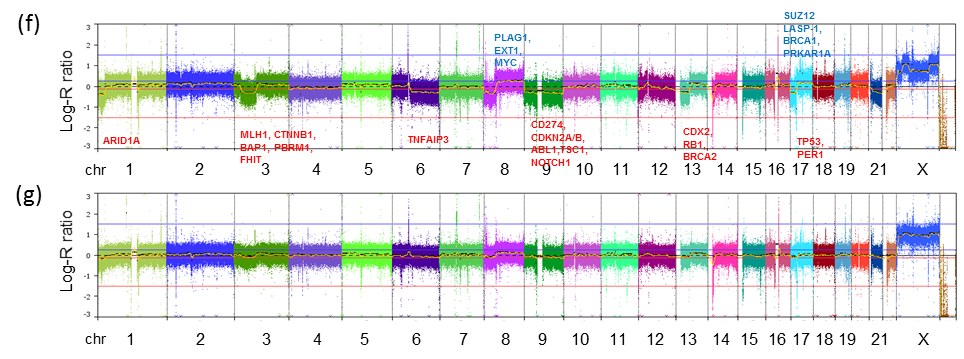


**Patient 3**

**Patient 1**

**Patient 2**

**Supplementary Figure 2**(a) Whole genome sequencing of Patient 2 reveals a focal CN loss at on chr. 9, overlapping cyclin dependent kinase inhibitor 2a (*CDKN2A*); (b) Whole genome sequencing of patient 3 ctDNA also shows focal chr. 9 copy number loss, which causes deletion of entire *CDKN2A* gene.

(a)

*CDKN2A*

(b)

**Supplementary Figure 3.** **(a)** Graph showing results of correlation analysis between 2-hydroxyglutarate and other identified metabolites (distance measure Pearson r) shows lactate is strongly correlated (correlation coefficient 0.74, p-value 0.003). **(b)** Hierarchical clustering of all compound features grouped patients and controls correctly. **(c)** Table shows correlation coefficients and associated statistics comparing identified metabolites with the orotic acid profile. **(d)** Box plots compare levels of most significantly altered metabolites between ICC patients and controls combining fold-change and p-value statistics (box plots: n=8 ICC patients, n=6 controls, boxes extend from the 25th to the 75th percentile with the median line in the middle. Whiskers are min to max with all data points shown).


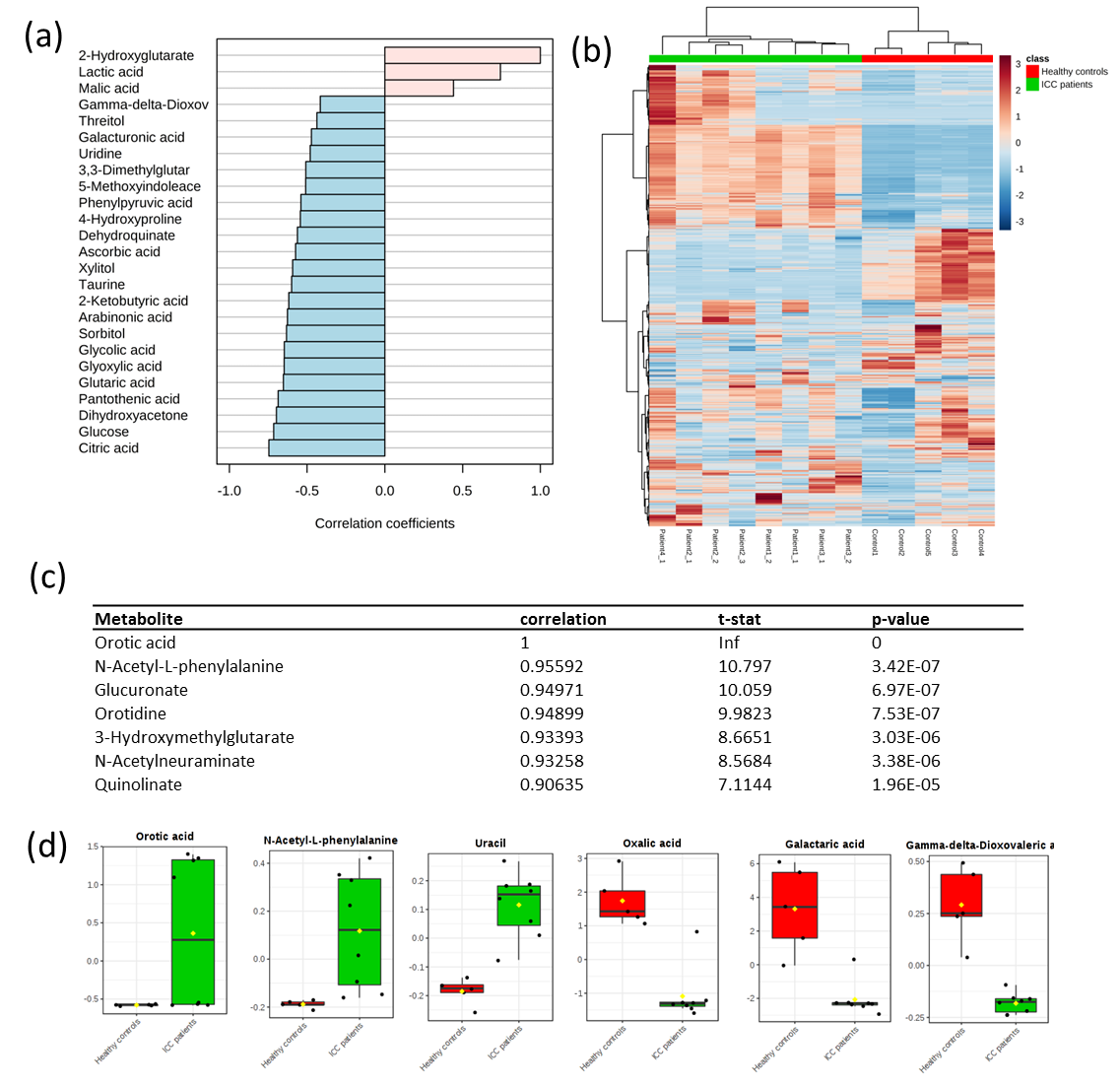


**Supplementary Figure 4**. Pyrimidine synthesis pathway (partial), showing enzymes involved in orotate synthesis and usage. CAD is a large, tri-functional enzyme comprised of Carbamoyl phosphate synthetase II, Aspartate transcarbamoylase and Dihydroorotase. UMPS (Uridine monophosphate synthase) is bi-functional and includes orotate phosphoribosyl transferase and orotidine monophosphate decarboxylase activities.


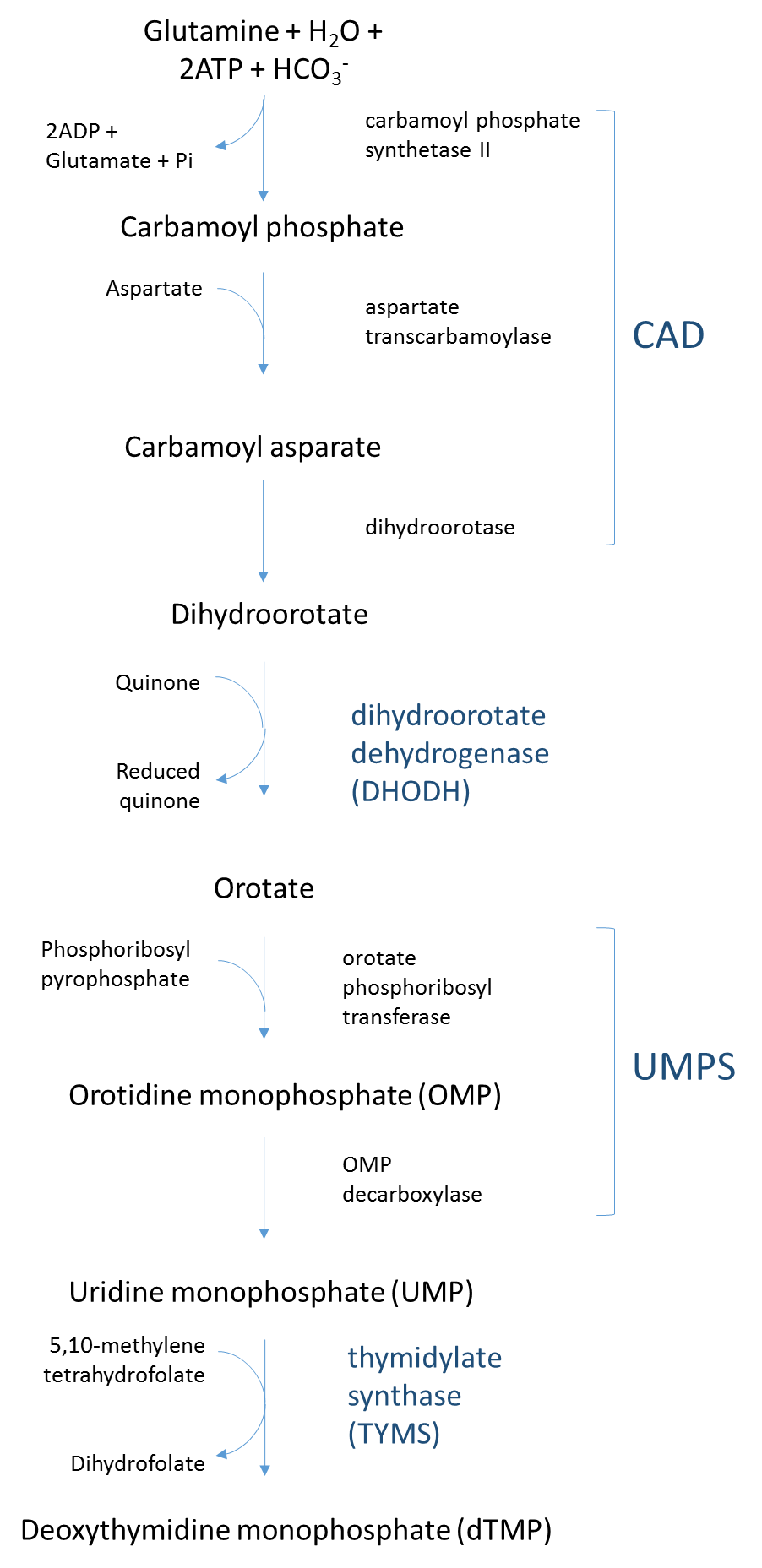


**Supplementary Table 1**. Patient information

| **Patient ID** | **Gender** | **Pathology** | **Overall survival from diagnosis**  **(months)** |
| --- | --- | --- | --- |
| 1 | female | well to moderately differentiated CK7 positive tumour | 24 |
| 2 | female |  | 15.5 |
| 3 | female | Positive immunostaining of tumour cells for CK8/18, CK19, CK7, AE1/AE3 and vimentin | 15 |
| 4 | male |  | 28 |

**Supplementary Table 2**. Cancer gene abbreviations list (used in text and Supplementary Figures 1-2)

| Gene symbol | Gene name (from http://cancer.sanger.ac.uk/census/) | Genomic location | Chr. band |
| --- | --- | --- | --- |
| ABL1 | v-abl Abelson murine leukemia viral oncogene homolog 1 | 10:26748570-26860863 | 10p11.2 |
| AKT1 | v-akt murine thymoma viral oncogene homolog 1 | 14:104770341-104792643 | 14q32.32 |
| ARID1A | AT rich interactive domain 1A (SWI-like) | 1:26696404-26780756 | 1p35.3 |
| BAP1 | BRCA1 associated protein-1 (ubiquitin carboxy-terminal hydrolase) | 3:52402288-52409878 | 3p21.31-p21.2 |
| BRCA1 | familial breast/ovarian cancer gene 1 | 17:43045678-43124096 | 17q21 |
| BRCA2 | familial breast/ovarian cancer gene 2 | 13:32316461-32398770 | 13q12 |
| CCDC6 | coiled-coil domain containing 6 | 10:59792917-59906424 | 10q21 |
| CCND2 | cyclin D2 | 12:4274041-4300009 | 12p13 |
| CD274 | CD274 molecule | 9:5456114-5467862 | 9p24 |
| CDK4 | cyclin-dependent kinase 4 | 12:57748525-57751717 | 12q14 |
| CDKN2A | cyclin-dependent kinase inhibitor 2A (p16(INK4a)) gene | 9:21968229-21974827 | 9p21 |
| CDX2 | caudal type homeo box transcription factor 2 | 13:27963115-27969006 | 13q12.3 |
| CTNNB1 | catenin (cadherin-associated protein); beta 1 | 3:41224069-41239342 | 3p22-p21.3 |
| EXT1 | multiple exostoses type 1 gene | 8:117799712-118111046 | 8q24.11-q24.13 |
| FGFR2 | fibroblast growth factor receptor 2 | 10:121479857-121593817 | 10q26 |
| FHIT | fragile histidine triad gene | 3:59752226-60536962 | 3p14.2 |
| GNAS | guanine nucleotide binding protein (G protein); alpha stimulating activity polypeptide 1 | 20:58891727-58910829 | 20q13.2 |
| HMGA2 | high mobility group AT-hook 2 (HMGIC) | 12:65825271-65963292 | 12q15 |
| HNF1A | HNF1 homeobox A | 12:120978769-121001192 | 12q24.2 |
| KRAS | v-Ki-ras2 Kirsten rat sarcoma 2 viral oncogene homolog | 12:25209795-25245384 | 12p12.1 |
| LASP-1 | LIM and SH3 protein 1 | 17:38870190-38918778 | 17q11-q21.3 |
| LIFR | leukemia inhibitory factor receptor | 5:38481595-38530647 | 5p13-p12 |
| MAF | v-maf musculoaponeurotic fibrosarcoma oncogene homolog | 16:79594460-79599902 | 16q22-q23 |
| MLH1 | E.coli MutL homolog gene | 3:36993548-37050653 | 3p21.3 |
| MYC | v-myc myelocytomatosis viral oncogene homolog (avian) | 8:127738263-127740958 | 8q24.12-q24.13 |
| NF1 | neurofibromatosis type 1 gene | 17:31095310-31374155 | 17q12 |
| NOTCH1 | Notch homolog 1; translocation-associated (Drosophila) (TAN1) | 9:136496071-136545786 | 9q34.3 |
| NOTCH2 | Notch homolog 2 | 1:119915306-120069406 | 1p13-p11 |
| NRAS | neuroblastoma RAS viral (v-ras) oncogene homolog | 1:114708535-114716160 | 1p13.2 |
| PBRM1 | polybromo 1 | 3:52548063-52679711 | 3p21 |
| PER1 | period homolog 1 (Drosophila) | 17:8141068-8150706 | 17p13.1-17p12 |
| PLAG1 | pleiomorphic adenoma gene 1 | 8:56166243-56168269 | 8q12 |
| PPARG | peroxisome proliferative activated receptor; gamma | 3:12351593-12434145 | 3p25 |
| PPHLN1 | Periphilin-1 | 12:42,326,125-42,448,620 | 12q12 |
| PRKAR1A | protein kinase; cAMP-dependent; regulatory; type I; alpha (tissue specific extinguisher 1) | 17:68515400-68530449 | 17q23-q24 |
| RAF1 | v-raf-1 murine leukemia viral oncogene homolog 1 | 3:12584514-12618721 | 3p25 |
| RB1 | retinoblastoma gene | 13:48303913-48480071 | 13q14 |
| SDHC | succinate dehydrogenase complex; subunit C; integral membrane protein; 15kDa | 1:161314406-161362433 | 1q21 |
| SUZ12 | suppressor of zeste 12 homolog (Drosophila) | 17:31937247-31999003 | 17q11.2 |
| TNFAIP3 | tumor necrosis factor; alpha-induced protein 3 | 6:137871228-137881319 | 6q23 |
| TP53 | tumor protein p53 | 17:7669609-7676594 | 17p13 |
| TSC1 | tuberous sclerosis 1 gene | 9:132896235-132928872 | 9q34 |

**Supplementary Table 3**. Sequencing results from circulating DNA of ICC patients including single nucleotide variants (SNVs) found by 50 gene cancer panel sequencing, and copy number (CN) losses and gains found using WGS, and SNVs found in four enzymes in pyrimidine metabolism pathway that affect orotate levels (*CAD*, carbamoyl-phosphate synthetase 2, aspartate transcarbamylase, and dihydroorotase (tri-functional enzyme); *DHODH*, dihydroorotate dehydrogenase; *UMPS*, orotate phosphoribosyltransferase and OMP decarboxylase (bi-functional enzyme); and *TYMS*, thymidylate synthase).

| **PERFORM study patient** | **Variant type** | **Variant** |  |  |
| --- | --- | --- | --- | --- |
| **Patient 1** | **ctDNA SNV from 50 cancer gene panel** | *NRAS* Q61R |  |  |
|  | **ctDNA CNV (baseline)** |  | **Chromosome region** | **additional notes on CNVs** |
|  |  | chr1 CN gain | chr1:118,850,518-121,365,903; chr1:143,645,493-170,484,920; chr1:202,216,392-203,451,046 chr1:203,465,043-248,487,849 |  |
|  |  | chr5 CN gain | chr5:1-39,527,977; chr5:91,489,662-101,928,452 |  |
|  |  | chr8 CN gain | chr8:46,838,816-85,175,888; chr8:85,255,095-146,364,022 |  |
|  |  | chr12 CN gain | chr12:1-34,854,262; chr12:38,228,473-39,200,964; chr12:41,614,666-44,024,182; chr12:52,379,417-70,627,186; chr12:71,219,927-72,011,929 |  |
|  |  | chr1 CN loss | chr1:203,451,047-203,465,042 |  |
|  |  | chr3 CN loss | chr3:1-198,022,430 | chr3 CN loss includes *UMPS* |
|  |  | chr6 CN loss | chr6:29,849,637-29,905,881; chr6:65,683,361-77,096,734; 6:78,484,854-171,115,067 |  |
|  |  | chr12 CN loss | chr12:39,200,964-41,614,665; chr12:44,024,208-44,246,330; chr12:47,469,632-52,379,416; chr12:70,627,187-71,219,926; chr12:72,012,005-133,841,829 |  |
|  |  | chr13 CN loss | chr13:19,347,376-115,169,878 |  |
|  |  | chr14 CN loss | chr14:19,827,829-107,289,458 |  |
|  |  | chr16 CN loss | chr16:56,353,960-90,354,753 | Chr16 CN loss includes *DHODH*, expect less orotate |
|  |  | chr17 CN loss | chr17:27,552,318-27,937,547; chr17:28,109,740-30,487,661 |  |
|  | **ctDNA SNV from WGS in pyrimidine metabolism pathway** |  | **Effect prediction scores for WGS variants** | **Structural observations** |
|  |  | none | none | none |
| **Patient 2** | **ctDNA SNV from 50 cancer gene panel** | *NRAS* Q61R |  |  |
|  | **ctDNA CNV (baseline)** |  | **Chromosome region** | **additional notes on CNVs** |
|  |  | chr2 CN gain | chr2:1-243,199,373 | chr2 CN gain includes *CAD* |
|  |  | chr5 CN gain | chr5:1-180,915,260 |  |
|  |  | chr10 CN gain | chr10:1-135,534,747 |  |
|  |  | chr20 CN gain | chr20:1-63,025,520 |  |
|  |  | chr3 CN loss | chr3:1-198,022,430 | chr3 CN loss includes *UMPS* |
|  |  | chr6 CN loss | chr6:80,337,558-95,471,735; chr6:95,565,209-171,115,067 |  |
|  |  | chr9 CN loss | chr9:1-141,213,431 | includes focal chr9p21.3 loss |
|  |  | chr17 CN loss | chr17:0-21,529,131 |  |
|  | **ctDNA SNV from WGS in pyrimidine metabolism pathway** |  | **Effect prediction scores for WGS variants** | **Structural observations** |
|  |  | *CAD,* NM_004341: c.G6343C; p. V2115L (germline) | CADD phred 13.7, DANN 0.85, REVEL 0.544 | May affect cooperative binding behaviour |
| **Patient 3** | **ctDNA SNV from 50 cancer gene panel** | *NRAS Q61R* |  |  |
|  |  | *IDH1 R132C* |  |  |
|  | **ctDNA CNV (baseline)** |  | **Chromosome region** | **additional notes on CNVs** |
|  |  | chr8 CN gain | chr8:40,463,321-42,066,437 |  |
|  |  | chr17 CN gain | chr17:25,263,161-81,195,210 |  |
|  |  | chr1 CN loss | chr1:1-27,786,538 |  |
|  |  | chr3 CN loss | chr3:24,040,862-81,544,211; chr3:157,054,246-157,186,371 | includes focal chr3p loss |
|  |  | chr6 CN loss | chr6:64,715,672-171,115,067 |  |
|  |  | chr9 CN loss | chr9:1-141,213,431 | includes focal chr9p21.3 loss |
|  |  | chr13 CN loss | chr13:19,020,006-52,129,307 |  |
|  |  | chr17 CN loss | chr17:0-21,532,007 |  |
|  |  | chr18 LOH | chr18:1-78,077,248 | includes *TYMS* gene |
|  | **ctDNA SNV from WGS in pyrimidine metabolism pathway** |  | **Effect prediction scores for WGS variants** | **Structural observations** |
|  |  | none | none | none |
| **Patient 4** | **ctDNA SNV from 50 cancer gene panel** | none |  |  |
|  | **ctDNA CNV (baseline)** |  | **Chromosome region** | **additional notes on CNVs** |
|  |  | chr17 CN gain | chr17:58,371,566-58,597,889 |  |
|  |  | chr1 CN loss | chr1:242,174,483-242,200,848 |  |
|  |  | chr13 CN loss | chr13:55,531,122-55,656,190; chr13:111,680,934-111,700,609 |  |
|  | **ctDNA SNV from WGS in pyrimidine metabolism pathway** |  | **Effect prediction scores for WGS variants** | **Structural observations** |
|  |  | *TYMS,* G5S (germline) | CADD phred 16.5, DANN 0.966, REVEL 0.049, M-CAP 0.046 | Very mild change unlikely to affect function |
|  |  | *CAD,* NM_004341: c.T274C; p. C92R | CADD phred 23.5, DANN 0.997, REVEL 0.869, M-CAP 0.483 | May interfere with allosteric regulation possibly resulting in gain of function |
|  |  | *CAD,* NM_004341: c.6139_6140del; p. D2047fs | Not available for frameshift | Removes aspartate transcarbamoylase functionality |
|  |  | *DHODH,* NM_001361: c.G1123T; p. A375S | CADD phred 19.29, DANN 0.932, M-CAP 0.055 | Surface mutation that may prevent K378 ubiquitination |

Links to Michelaɴɢʟo 3D annotation results for

CAD: <https://michelanglo.sgc.ox.ac.uk/r/cad>

DHODH: <https://michelanglo.sgc.ox.ac.uk/r/dhodh>

**Supplementary Table 4**. Full list of identified metabolites and their raw extracted ion chromatogram (EIC) peak areas from all samples.

**Supplementary Table 5.** Gene information of cancer gene panel (sourced from ThermoFisher Scientific, separate Excel table).
